# Supplementary material for: Ferroelectric freestanding hafnia membranes with metastable rhombohedral structure down to 1-nm-thick
Source: Nat Commun. 2024 Jun 25;15:4789. doi: 10.1038/s41467-024-49055-w (PMC11199652; doi:10.1038/s41467-024-49055-w)
Supplement: Supplementary file 1 — Supplementary Information [file 41467_2024_49055_MOESM1_ESM.pdf]

Supplementary Materials for

Ferroelectric freestanding hafnia membranes with metastable rhombohedral structure down to 1-nm-thick

Yufan Shen, Kousuke Ooe, Xueyou Yuan, Tomoaki Yamada, Shunsuke Kobayashi, Mitsutaka Haruta, Daisuke Kan*, Yuichi Shimakawa

Corresponding author: dkan@scl.kyoto-u.ac.jp

The PDF file includes:

Supplementary Text: Sections 1-9
Figs. S1 to S14
Tables S1 and S2.
References in Supplementary Materials

Supplementary Text

Section 1. Thickness, surface topography, and crystallinity of epitaxial HZO thin films

The thicknesses of the HZO epitaxial layers grown on (La,Sr)MnO_3-bufferred $SrTiO_3$ substrates (before exfoliation processes) were evaluated by X-ray reflectivity (XRR) measurements. The XRR profiles for the HZO layers whose thickness was set to be 1, 3, and 5 nm are shown in Fig. S1(A). The observed features in the profiles are well reproduced by assuming the HZO thickness as designed.

Fig. S1(B) shows the AFM surface topographies of 1, 3, and 5-nm-thick HZO epitaxial films. All films have a step-and-terrace surface topography, which is essentially the same as those for the STO substrates. The root mean square (RMS) roughness is also low, 1.09 Å, 1.29 Å, and 1.21 Å for the 1, 3, and 5-nm-thick films, respectively. These observations indicate that the HZO films are of good quality.

Furthermore, the crystallinity of the epitaxial thin films was evaluated from omega scans of the (111) HZO reflections. The results are shown in Fig. S1(C). It can be seen that the half-width full maximum (HWFM) of the (111) reflections for all epitaxial films studied here are as low as 0.05°, ensuring the crystallinity of our HZO epitaxial films.

Section 2. Ferroelectric properties of epitaxial HZO thin films

Fig. S2 shows the room-temperature polarization versus electric field hysteresis (P-E) loop for the 1, 3, and 5-nm-thick HZO epitaxial films. In the loops measured at 1, 10, and 100 kHz for 5-nm-thick thin films (Fig. S2(A)), clear polarization switching with respect to the electric field can be observed. Furthermore, the ferroelectric switching current can also be seen in each current versus electric field (I-E) loop, confirming ferroelectricity in the HZO epitaxial films. We note that as the measurement frequency is increased, the leakage current contribution is further reduced. The $2P_r$ obtained from the P-E loops measured at 100 kHz is 20 μC/cm^2, which is almost the same as $2P_r$ obtained from the 1 kHz and 10 kHz loops (24 μC/cm^2). Furthermore, these 2Pr values are slightly larger than the ones obtained from PUND measurements (19 μC/cm^2, Fig.1(C)). These results indicate that the leakage current contribution in P-E loops is less significant and that the non-saturated P-E loops probably result from paraelectric components co-existing in the films, for example, grains with the monoclinic structure. The slight horizontal shift in the loops might originate from the asymmetric electrostatic boundary between the top electrode/HZO and the bottom electrode/HZO interfaces (the top and bottom electrodes are Au and LSMO, respectively). It should be noted that the observed large E_c (5 MV/cm, 1 kHz) is comparable with previously reported values of E_c for epitaxial thin films with similar thicknesses[1]. In addition, no wake-up effects are observed.

On the other hand, the 1 and 3-nm-thick epitaxial films are too leaky to evaluate switching polarization from P-E loop and PUND measurements as shown in Figures S2 (B-D), and it can be seen that the P-E loops were heavily contaminated with leakage currents. The switching polarization (2Pr), determined based on the assumption that the leakage currents equally contribute to the charges measured under the P(N) and U(D) voltage pulses in PUND measurements, also keeps increasing with electric fields (Fig. S2(D)), indicating that leakage currents in the 1 and 3-nm-thick epitaxial films are very large and that eliminating the leakage current contribution is difficult even for PUND measurements.

The ferroelectric domains for the 1-nm-thick epitaxial HZO thin film were also characterized with the PFM system (Fig. S3). The film displays a single-domain structure with polarizations all pointing downward. We also performed box-in-box domain writing and confirmed that the written

box-in-box domains with polarizations pointing to opposite directions still remained 30 minutes after the domain writings (Fig. S3), indicating that the films have robust ferroelectricity.

Section 3. Thickness verification for HZO membranes

After the exfoliation and transfer processes, the thickness of the exfoliated HZO membranes was confirmed from the height profiles across the edges of the membranes obtained by AFM. Figures S5(A) and S5(B) show AFM surface topographies and height profiles for HZO membranes that were fabricated by exfoliating 3- and 1-nm-thick HZO epitaxial films. For both membranes, height steps are observed when going from the substrates to the membranes, and the magnitudes of these steps are essentially the same as the thicknesses of the original epitaxial films. The height jumps at the membrane edges possibly stem from either curling or bending of the membranes, which often occurs in two-dimensional materials transferred with dry transfer methods[2,3].

Furthermore, to cross-check the thickness of the membranes, we evaluated the thickness of the thinnest (1-nm-thick) membrane by using the electron energy loss (EEL) spectrum. Fig. S5(C) shows the zero loss peak (ZLP) EEL spectrum obtained from the HZO membrane. To clearly see the plasmon intensity, the ZLP data in Fig. S5(C) are re-plotted on the semi-logarithmic scale in Fig. S5(D). The relative thickness, t/λ, was calculated to be 0.0189. The λ value for $Hf_{0.5}Zr_{0.5}O_2$ is 82.28 nm, giving the thickness t of 1.55 nm. As shown in Fig. S5(E), a slight signal at the C K-edge indicates the presence of minor contamination in the membrane sample, likely due to electron beam irradiation and sample handling. Therefore, the obtained thickness (1.55 nm) would be overestimated and slightly larger than the actual thickness. These observations imply that, during the exfoliation and transfer processes, the HZO layers remain intact and maintain their thicknesses.

Section 4. Elementary mapping and stability of the orthorhombic phase in HZO membranes

Cross-sectional STEM-EDS elementary mappings were conducted on the 5-nm-thick HZO membranes (Fig. S6(A)). It should be noted that the atomic columns in the membranes can not be spatially resolved because the HZO domains overlap along the observing direction and misalignment of the incident electron beam to the domains. The Hf and Zr EDS signals were detected in going from the SiO_2/Si substrate to the membrane region, which is accompanied by the disappearance of the Si signals from the substrate. This observation signifies the successful transfer of the membranes. Weak Mn signals were also detected inside the HZO membranes, as confirmed by the EDS spectra in Fig. S6(B-E), while no signals from La and Sr were found. The Mn diffusion in the HZO region probably stems from interfacial redox reactions during epitaxial growth of the thin films[4].

The stability of the metastable orthorhombic phase in HZO was also examined by making XRD measurements. Figure S7 shows the patterns of the 5-nm-thick HZO membranes three and nine weeks after exfoliation. Apparently, no changes in the diffraction profiles around the (111) reflection from HZO occurred during these periods, which indicates the stability of the metastable phase of HZO. It should be noted that a broad peak in the 2θ region around 22° originates from a sample holder used in our XRD measurement system.

Section 5. Structural phase identification and distribution in ultrathin HZO membranes

Figure S8 shows the identified phases and their distribution in the 1, 3, and 5-nm-thick HZO membranes. The atomic arrangements in the plan-view HAADF-STEM images of the identified phases and their FFT patterns (Fig. S8(A)) are well reproduced by the (111)-oriented monoclinic (i.e., the m-(-111)) structure, the (111)- and (001)-oriented orthorhombic (o-(111) and

o-(001)) structures, or the (111)-oriented rhombohedral structure (r-(111)). Moreover, this phase identification is further checked by comparing with the FFT patterns simulated from polymorph structures of HZO and their atomic arrangements shown in Fig. S8(B). It can be concluded that the structural phases of grains in our HZO membranes are identified as r-(111), o-(001), o-(111), or m-(-111). Our structural phase distribution analysis over larger observation areas (90 x 90 nm) indicates that the 1-nm-thick membranes have a majority of r-(111) grains and a minority of o-(001) and m-(-111) grains (Fig. S8(C)). When the thickness is increased to 3 nm, in addition to a minority of o-(001) grains, an amount of m-(-111) grains slightly increases, while the r-(111) grains still dominate in the membranes, as shown in Fig. S8(D). We note that the minor grains categorized as o-(001) in the 1 and 3-nm-thick membranes show almost no periodic modulation in the distance between neighboring Hf/Zr atoms characteristic of the o-(001) structure, in contrast to those in the 5-nm-thick membrane. The observed cation arrangements imply that structure candidates of the (001)-oriented grains in the 1 and 3-nm-thick membranes are cubic, tetragonal, and rhombohedral. However, as shown in Fig. S8b, cation arrangements of these structures projected in the [001] direction are essentially the same, and identifying the structural phase of the (001)-oriented grains in the 1 and 3-nm-thick membranes requires further characterizations. We note that this sorting of the minor domains in the 1 and 3-nm-thick membranes does not change our main conclusion. On the other hand, the 5-nm-thick HZO film is dominated by grains with the o-(111) structure, with a mixture of grains having the o-(001) and m-(111) structures (Fig. S8(E)). The presence of the most stable m-(-111) grains as increasing the thickness implies a thickness-induced structural relaxation. A similar thickness-induced structural relaxation associated with the formation of the monoclinic phase was reported for epitaxial HZO thin films[1]. Moreover, exfoliating HZO epitaxial layers from the substrates would relax the strain accumulated in the HZO, and thus, the exfoliation process might facilitate a transformation of the metastable phases of HZO into the most stable monoclinic phase.

We also note that while a tiny amount of amorphous grains (< 4%) are detected in the 1-nm-thick membranes (indicated in pink in Fig. S8(C)), no amorphous grains are observed for 3- and 5-nm-thick membranes. These observations ensure the crystallinity of the HZO membranes.

Fig. S9 displays simulated SAED patterns for the m-(111), o-(111), o-(001), and r-(111) structures and the experimentally observed SAED patterns for the 1- 3- and 5-nm-thick HZO membranes. The diffraction patterns of each structural phase observed for the membrane samples are different. Rotating and superimposing these different patterns in a series of angles with a step as $\Delta\varphi$ perfectly reproduce the experimentally observed SAED patterns for 1-nm-, 3-nm- and 5 nm-thick membranes, including weak diffraction spots (Figs. S9(B)-(C), S9(D)-(E), and S9(F)-(G), respectively). This is consistent with the results of the structural phase distribution analysis in Fig. S8. The intensity profiles are plotted against the reciprocal distance of the experimental SAED patterns in Fig. S10. The main peaks marked by the stars and triangles for the 1, 3, and 5 nm-thick HZO membranes match the ones simulated with the rhombohedral and orthorhombic structures. It should be noted that distinguishing between the orthorhombic and monoclinic phases might be difficult because some diffraction spots from these phases, for example, Orthorhombic 202 and Monoclinic 022 spots, appear at almost the same position. Similarly, distinguishing between Orthorhombic 022 and Rhombohedral 220 is also difficult. Nonetheless, the cation arrangements of these structural phases totally differ, and these phases are distinguishable from the high-resolution HAADF-STEM images. The lattice constants of the rhombohedral and orthorhombic phases in the membranes are close to those used in the simulations. For the orthorhombic phase,

the lattice parameters are *a*=5.04Å, *b*=5.07Å and *c*=5.27Å. For the rhombohedral phase, they are *a*=5.05Å and α=89.98° (Table. S1).

Section 6. Imprint effect on HZO membranes

PFM characterizations were used to check for imprint effect in the ultrathin HZO membranes. The membrane samples were placed on Pt/Au sputtered SiO_2/Si substrates (Fig. S11(A)). The samples were polled with a sequence of gradually changing square bias pulses, as depicted in Fig. S11(B), and the out-of-plane piezoelectric response was measured between bias pulses (off-field piezoelectric response).

Previously, the imprint effect in HZO thin films was shown to be related to the charge injections and traps, which can be visualized by using poling pulses with different sweeping directions[5]. As shown in the PFM phase hysteresis loops (Fig. S11(B)), upon changing the sweeping directions of the poling pulses, the loops do not show any changes depending on the bias sweep direction, signifying that the imprint effect due to charge injections are negligible in the HZO membranes. Furthermore, cycling hysteresis loop measurements (Fig. S11(C-E)) result in no apparent changes in the loops, implying that charge injections and their effect on ferroelectric and piezoelectric properties, like imprint effect, are negligibly small in the HZO membranes.

Section 7. Ferroelectric domain writing in HZO membranes

Figure S12 summarizes the results of the PFM observations of the ferroelectric domains in the HZO membranes. The 1-nm-thick membrane shows a uniform single-domain structure (Fig. S12(A)), similar to what was observed in the epitaxial thin films (Fig. S3). On the other hand, the 3-nm-thick membrane shows a mosaic ferroelectric domain pattern (Fig. S12(B)). The 1-nm-thick HZO membranes have the rhombohedral crystal structure rather than the orthorhombic one of the thicker HZO membranes, implying that the crystal structure affects the ferroelectric domain pattern in HZO membranes. Similar ferroelectric domain evolution associated with structural changes was previously reported to occur in ultrathin membranes of related oxides like $BiFeO_3$[6].

As shown in Fig. S12(C), the ferroelectric domain in the 3-nm-thick HZO membranes can be switched by applying ±8V d.c. bias through PFM. This shows that the exfoliated HZO membranes have ferroelectricity.

Section 8. PUND Measurements of HZO membranes

We performed PUND measurements with sequences of square voltages having pulse widths of 25 μs and evaluated the switching polarizations in the HZO membranes (Fig. S13(A)). Fig. S13(B-D) shows typical charge changes induced by the PUND voltage sequences in 1-, 3-, and 5 nm-thick HZO membranes fabricated into cross-bar junctions. Prior to the measurements, the polarization of the HZO membranes was pre-set to align along one direction by applying positive voltage pulses (labeled as U). The recorded charges (electric displacements) are labeled as Q1. The polarization is then flipped by applying a negative voltage pulse (N); the charges collected after this switching are marked as Q2. We should note that, in addition to the polarization switching, leakage current also contributes to Q2. Therefore, the charge difference resulting from the first voltage pulse (Q2−Q1) consists of both polarization switching and leakage current. To extract the contribution from the polarization switching, we applied a second downward voltage pulse (D) with the same amplitude as the previous negative pulse (N) and measured the charges Q3. Because the difference between Q2 and Q3 is supposed to come from only the leakage current, the switchable (ferroelectric) polarization can be evaluated by taking the difference [(Q2−Q1) −

(Q3−Q2)] = $2P_r$. The polarity of the extracted polarization was defined in accordance with the direction of the setting pulse; i.e., a negative (positive) pre-setting pulse enables the positive (negative) polarization to be extracted.

We note that the polarization calculation described above assumes that the leakage currents equally contribute to the charges measured under the P(N) and U(D) voltage pulses. However, this assumption might not be correct when leakage currents are large. Therefore, to eliminate these non-ferroelectric (leakage current) contributions and determine polarization values from PUND measurements more precisely, we estimated the non-ferroelectric contribution from charge profiles measured with the N and D voltage pulses, and corrected the 2Pr values that were calculated as $2P_r$ = [(Q2−Q1) − (Q3−Q2)]. The charges originating from leakage current flow while rectangular electrical pulse was applied (Δt = 25 μs) can be calculated as $Q_{\mathrm{leakage}} = \int I_{\mathrm{leakage}}dt$, in which I_{leakage} should be constant during the voltage pulse application and can be obtained as the slope of charge profiles (indicated with purple dot lines in the right panel of Fig. S13(A)). To extract I_{leakage} associated with each voltage pulse application (P, U, N, and D pulses), the charge profiles in the time region between 15 and 25 μs after each voltage pulse was applied were linear-fitted and Q_{leakage} was calculated as $Q_{\mathrm{leakage}} = |I_{\mathrm{leakage}}|\cdot\Delta t$ where Δt = 25 μs. The results are summarized in Table. S2. The difference in the leakage current contribution between the P and U (or N and D) pulses are thus estimated as $\Delta Q_{\mathrm{leakage}} = (|I_{\mathrm{leakage\text{-}P}}|-|I_{\mathrm{leakage\text{-}U}}|)\cdot\Delta t$ (or $= (|I_{\mathrm{leakage\text{-}N}}|-|I_{\mathrm{leakage\text{-}D}}|)\cdot\Delta t$). If the leakage current would contribute to 2Pr largely, we should see a significant amount of $\Delta Q_{\mathrm{leakage}}$. However, $\Delta Q_{\mathrm{leakage}}$ in our measurements is estimated to be at most 6 μC/cm^2, which is much smaller than the observed 2Pr (around 30 μC/cm^2 before the correction), indicating that the leakage current contribution is less significant. The 2Pr values plotted in the revised version, except for the ones in Fig. S2(D), are all corrected for the leakage current contribution based on this approach. We also note that the charge profiles during PUND measurements are consistent over repeated measurements (Figs. S13(E-F)), and the 2Pr values are steady against the measurement cycles (Fig. S13(G)), further validating that the non-ferroelectric contribution in our transferred membranes is less important.

In addition, we repeated the PUND measurements on multiple cross-bar junctions (Fig. S14) and confirmed the reproducibility of the ferroelectric polarizations for the 1-nm-thick HZO membranes. We also confirmed that the E_s values of all the 1-nm-thick membranes characterized here are large, over 10 MV/cm, indicating that a large E_s is intrinsic to HZO in the ultrathin limit. The large E_s might stem from changes in the polarization switching manner from three-dimensional switching for thicker HZO to two-dimensional lateral switching for thinner HZO.

Supplementary Figures:

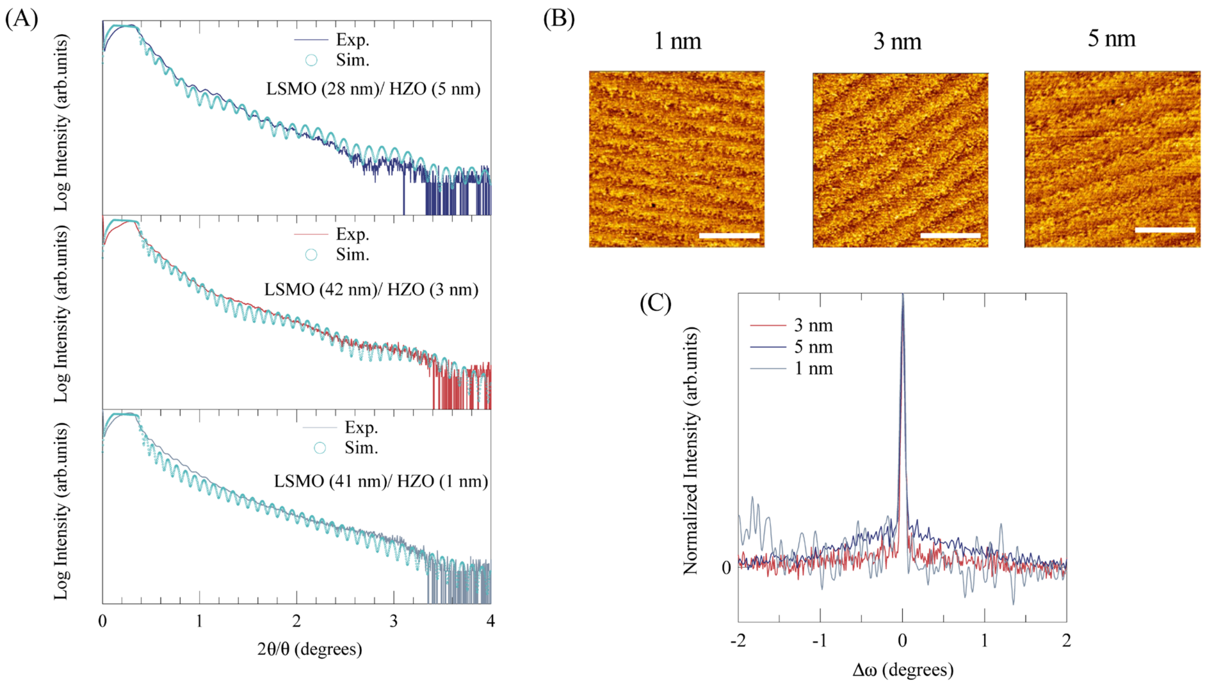

Fig. S1. Thickness measurements, surface morphology, and crystallinity of HZO epitaxial films. (A) Experimental and simulated XRR patterns of the 1-, 3, and 5-nm-thick HZO epitaxial thin films. (B) AFM surface morphologies of 1-nm, 3-nm, and 5-nm-thick HZO epitaxial films. The scale bars in the figures correspond to 1 μm. (C) Omega scan results for the 1, 3, and 5-nm-thick HZO epitaxial films. The 2θ values were set to 26.9°, 29.1° and 29.7° for the 1, 3, and 5-nm-thick films, respectively.

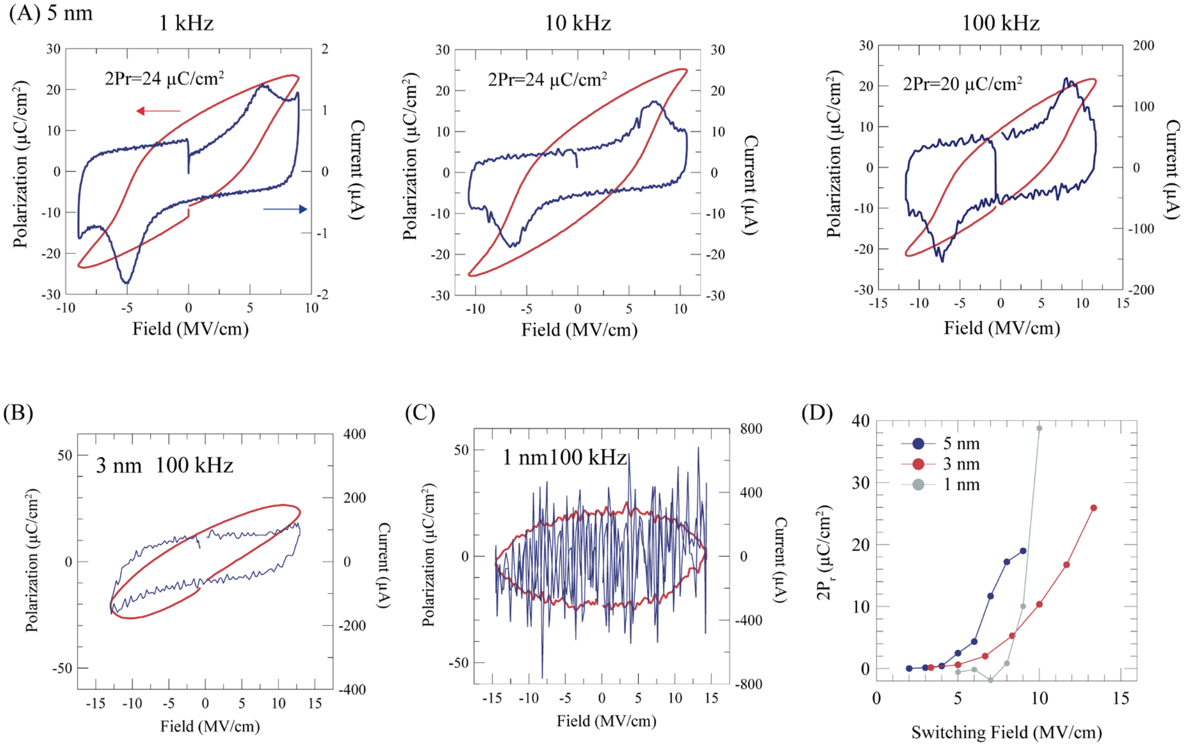

Fig. S2. P-E and I-E loops of epitaxial HZO thin film. (A) P-E and I-E loops of 5-nm-thick epitaxial HZO thin film measured at 1 kHz, 10 kHz, and 100 kHz. (B-C) P-E and I-E loops of (B) 3-nm- and (C) 1-nm-thick epitaxial HZO thin film measured at 100 kHz. (D) PUND results of the 1, 3, and 5-nm-thick HZO epitaxial films. Note that the 2Pr values plotted are determined based on the assumption that the leakage currents equally contribute to the charges measured under the P(N) and U(D) voltage pulses (the 2Pr values determined by conventional PUND measurements).

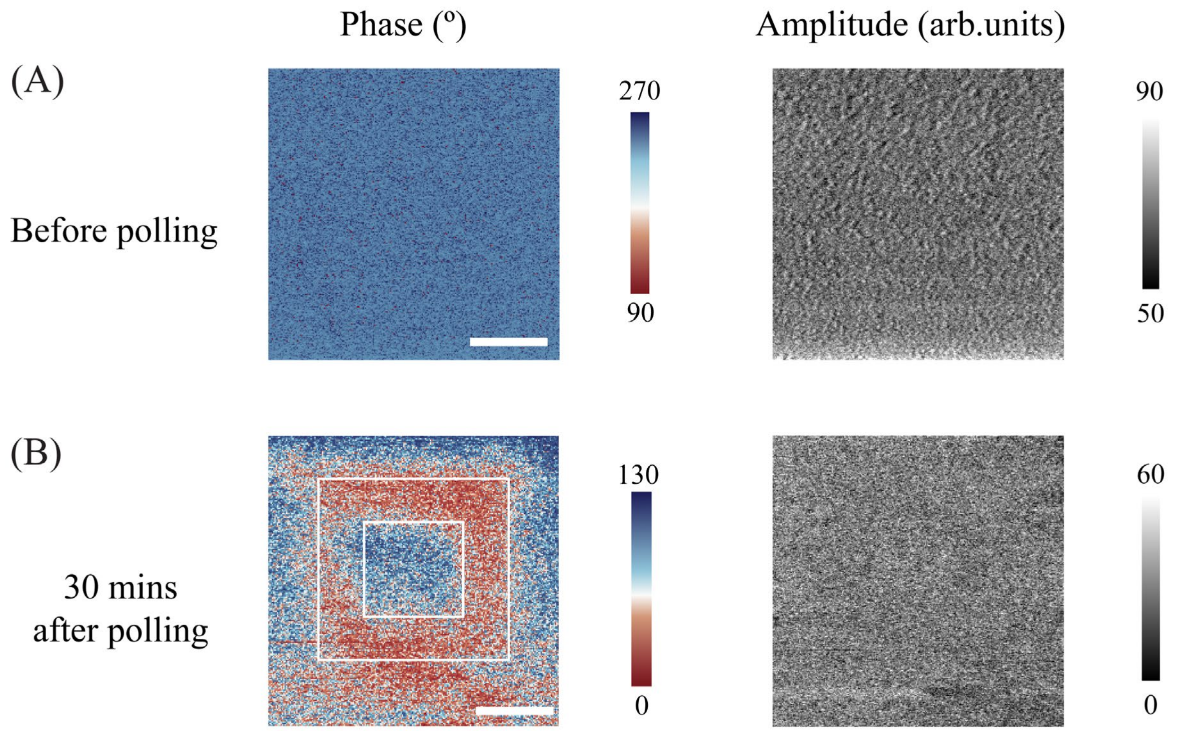

Fig. S3. Ferroelectric domain mapping of the 1-nm-thick epitaxial HZO thin films. (A) Before poling (as-grown state) and (B) 30 mins after poling. The poling bias is ±2 V. The scale-bars in the figures denote 500 nm.

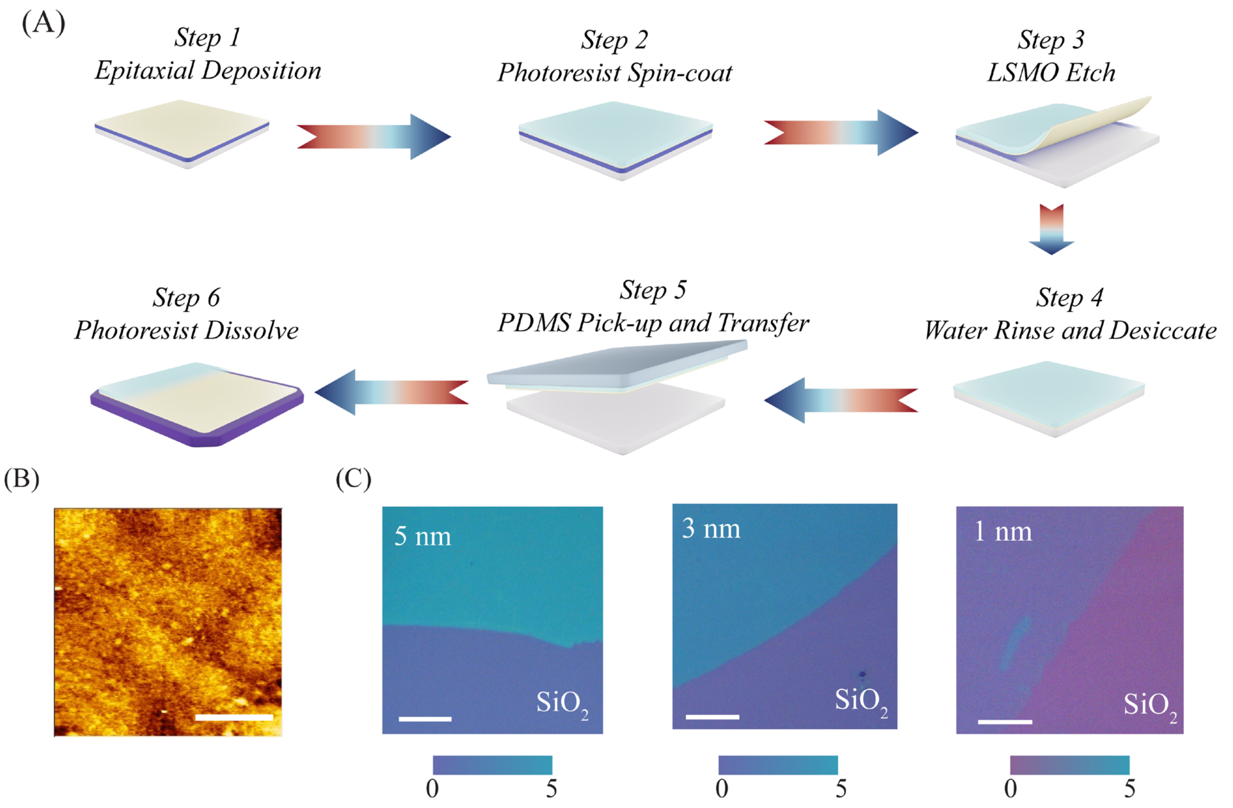

Fig. S4. Fabrication process and surface morphology of ultrathin HZO membranes. (A) Detailed fabrication processes of HZO membranes. (B) AFM surface morphology of 3-nm-thick HZO membranes. The scale bar in the image denotes 1 µm. (C) Optical images of 1-, 3- and 5-thick HZO membranes transferred onto SiO_2/Si substrates. The color bars below indicate the thickness in nanometers. The scale bars in the images denote 10 µm.

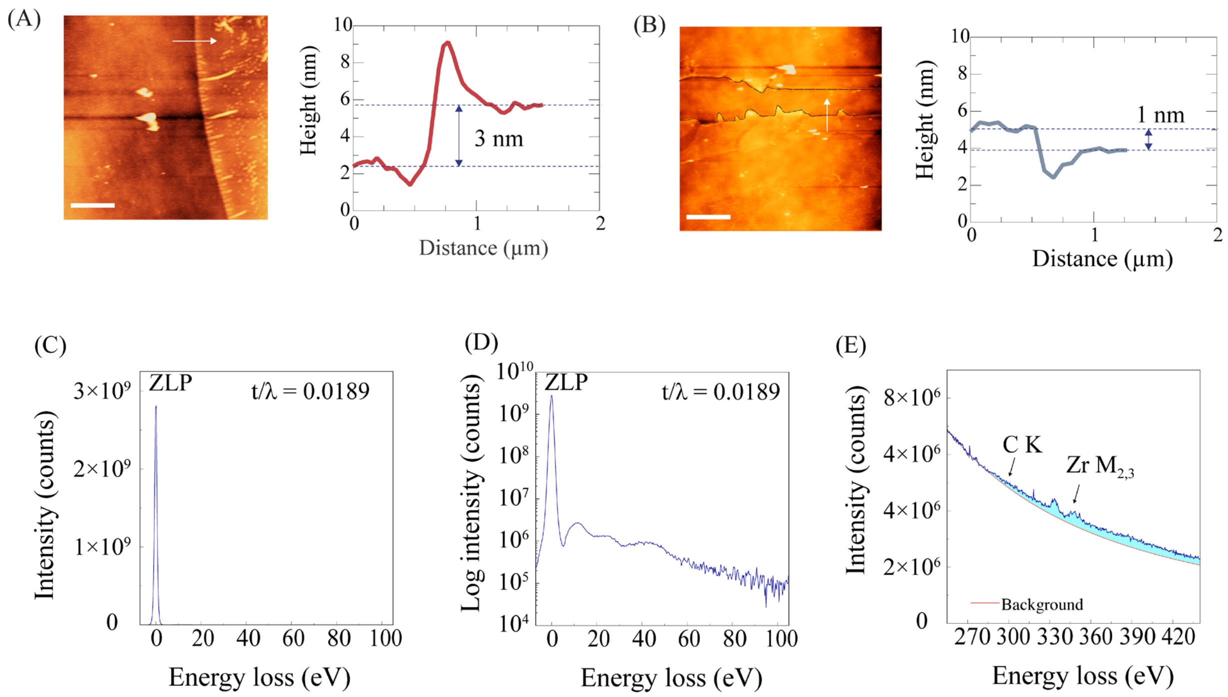

Fig. S5. Thickness confirmation for HZO membranes. (A-B) AFM surface topography of (A) 3-nm-thick and (B) 1-nm-thick HZO membranes. The height profile was extracted along the white arrow in the corresponding morphologies. The black dotted line in (B) indicates the edge between the SiO_2/Si substrate and the 1-nm-thick HZO membrane. The scale bars in (A) and (B) denote 2 μm. (C) ZLP EEL spectrum obtained from the 1-nm-thick HZO membrane. (D) ZLP EEL spectrum from (C) on the semi-logarithmic scale. (E) EEL spectrum near the C K-edge of the 1-nm-thick HZO membrane.

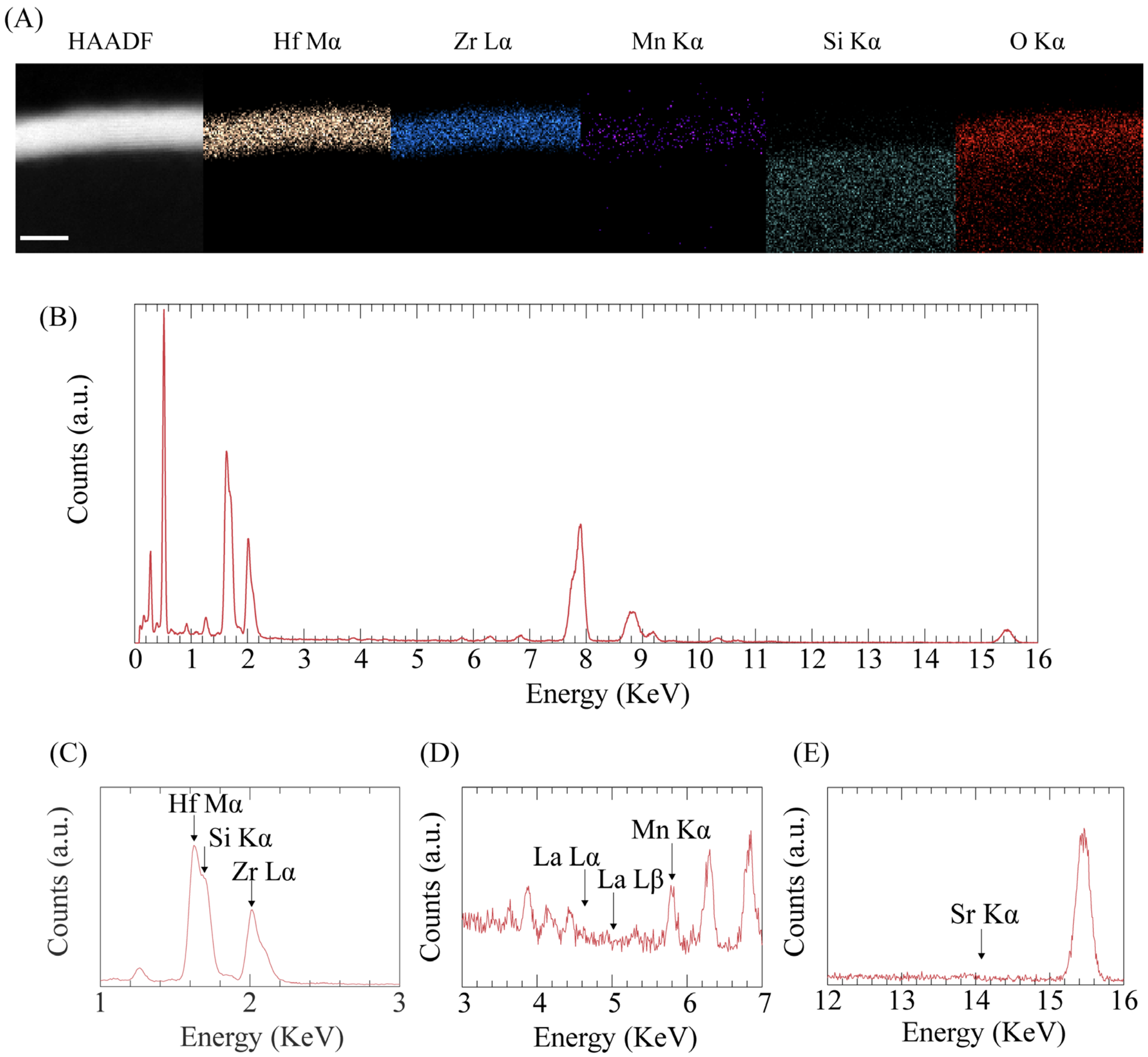

Fig. S6. Cross-sectional STEM observation and elementary mapping of 5-nm-thick HZO membranes. (A) Cross-sectional STEM-EDS elementary mapping of 5-nm-thick HZO membranes transferred onto SiO_2/Si substrates. The scale bar in the figure denotes 5 nm. (B-E) EDS spectra for 5-nm-thick HZO membranes. (C), (D) and (E) are magnified spectra in the 1-3 keV, 3-7 keV and 12-16 keV regions in (B), respectively.

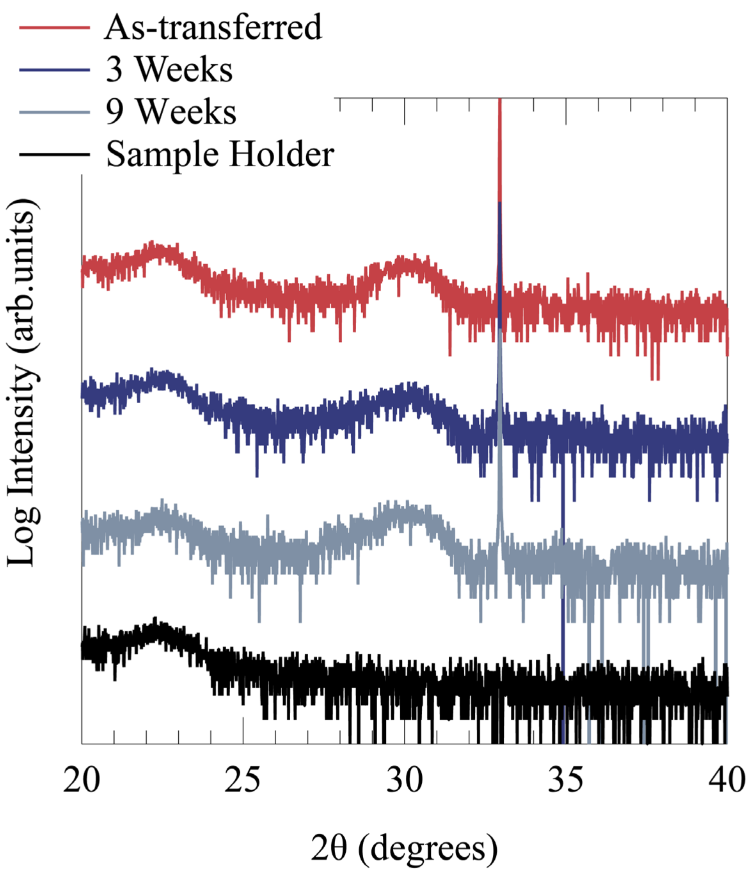

Fig. S7. XRD patterns of the 5-nm-thick HZO membrane that were measured at as-transferred, 3 weeks after transfer, and 9 weeks after transfer. The broad peak seen in 2θ ~ 22° comes from the sample holder used in our XRD measurements.

(A)

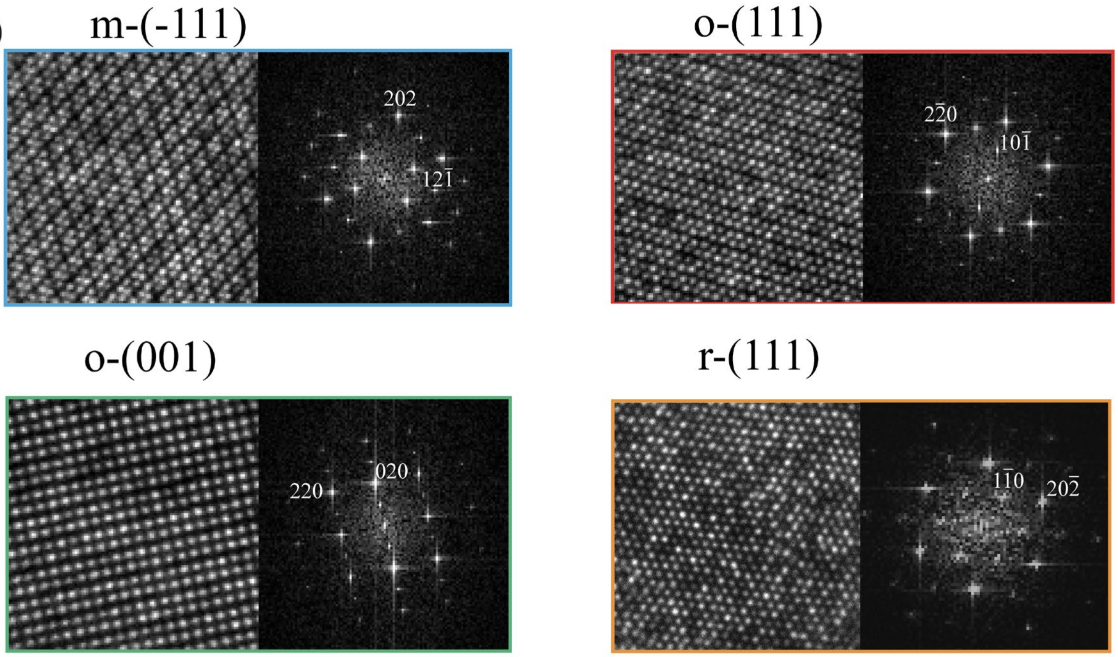

m-(-111)
202
12$\bar{1}$
o-(111)
2$\bar{2}$0
10$\bar{1}$
o-(001)
220
020
r-(111)
1$\bar{1}$0
20$\bar{2}$

(B)

	001	101	111
Cubic	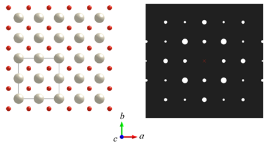	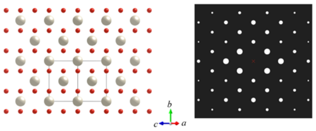	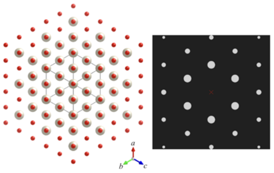
Tetragonal	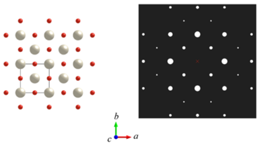	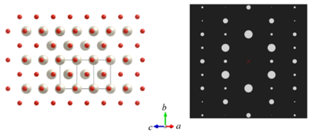	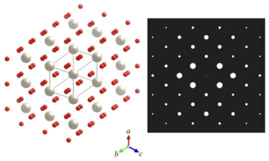
Rhombohedral	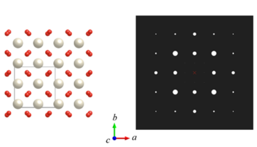	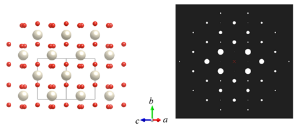	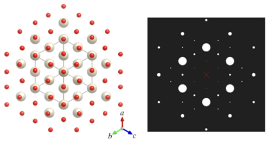
Orthorhombic	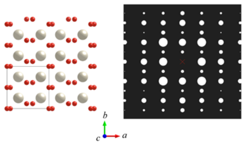	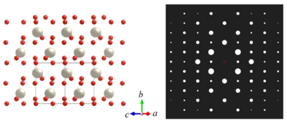	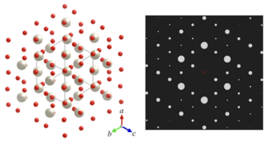
Monoclinic	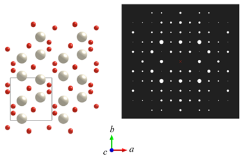	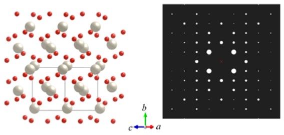	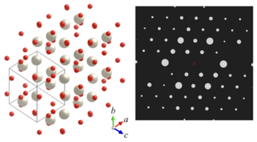

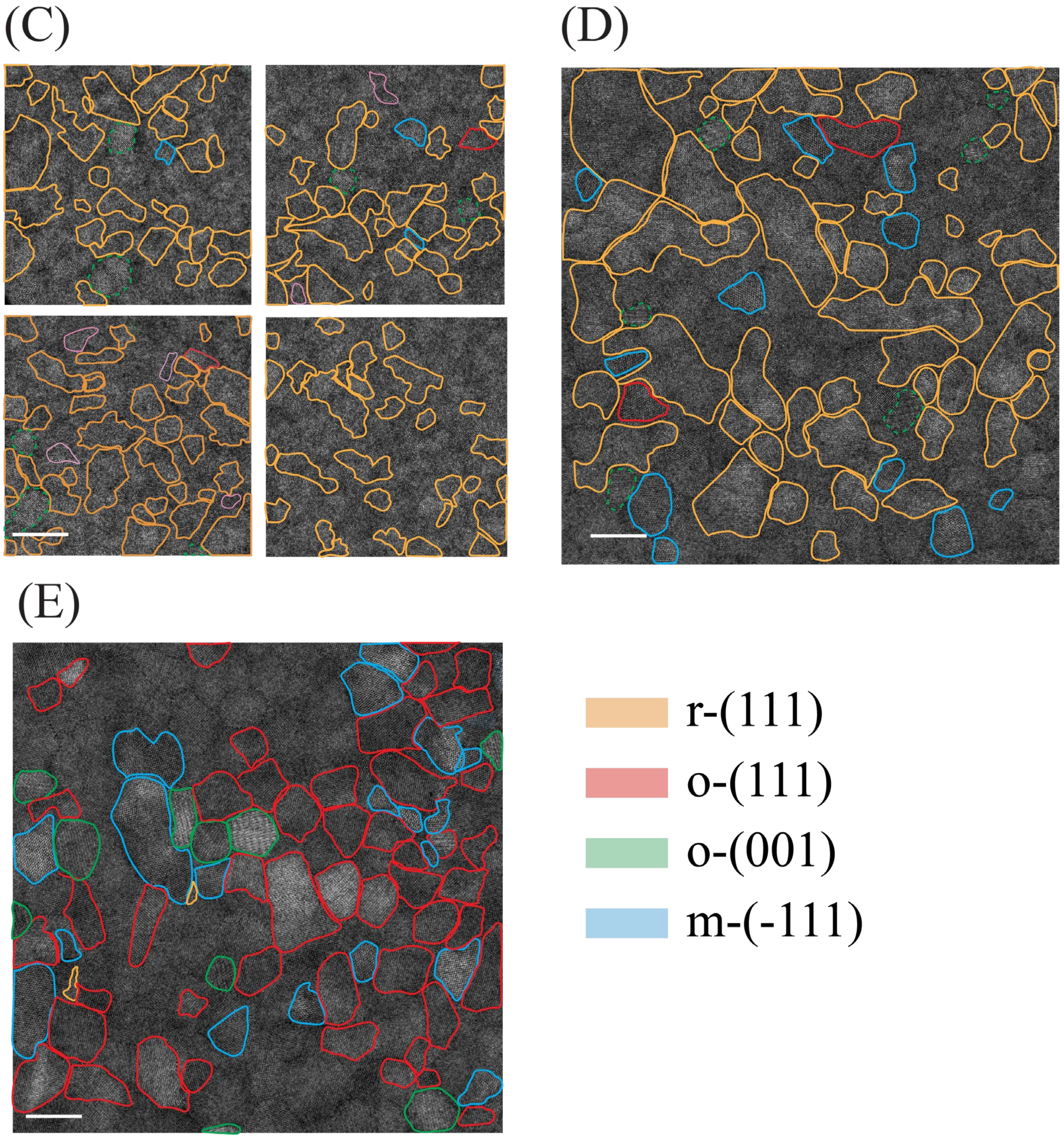

Fig. S8. Phase distribution in HZO membranes. (A) Plan-view HAADF-STEM images and their FFT patterns for the m-(-111), o-(111), o-(001) and r-(111) structures of HZO. (B) Atomic models of polymorph structures of HZO and their simulated FFT patterns. The model shown as the monoclinic 111 is the one observed along the [-111] direction of the monoclinic structure. (C-E) Plan-view HAADF-STEM images and structure phase distribution in (C) 1- and (D) 3- and (E) 5-nm-thick HZO membranes. The scale bars in (C-E) correspond to 10 nm. The grains having the r-(111), o-(111), o-(001), and m-(-111) atomic arrangements are colored orange, red, green, and blue, respectively. The (001)-oriented grains circled with dashed green lines in (C) and (D) show almost no periodic modulation in the distance between neighboring Hf/Zr atoms characteristic of the o-(001) structure. The amorphous grains are colored pink. Note that the amorphous grains are

found only in the 1-nm-thick membrane, not in the 3 and 5-nm-thick membranes. The uncolored grains are ones whose structural phases cannot be identified because the atomic columns are not well resolved because of misalignment between each grain and the incident electron beam.

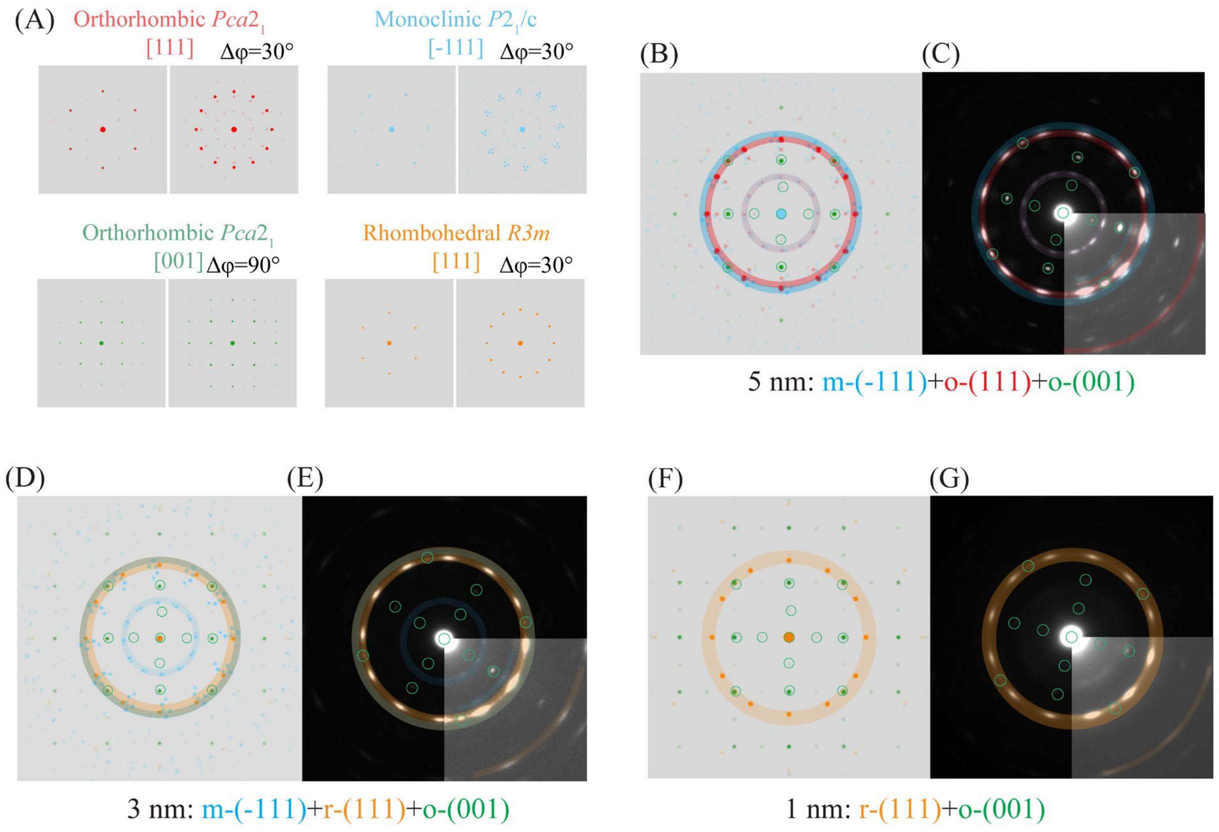

Fig. S9. (A) Simulated and (B-G) experimentally observed SAED patterns for (B-C) 5-nm-, (D-E) 3-nm-, and (F-G) 1-nm-thick HZO membranes. (A) Simulated SAED patterns for the (111)- and (001)-oriented orthorhombic, (-111)-oriented monoclinic and (111)-orientated rhombohedral structures of HZO, the diffraction patterns have the same color codes as in (A) to identify which diffraction patterns originate from which structural phases of HZO. The inset in (C), (E), and (G) show the high-contrast parts of the SAED. For the 1 and 3-nm-thick membranes, the diffraction spots of the o-(001) structure with the smallest reciprocal space distance are hardly seen. This is probably why the (001)-oriented grains in these membranes have negligibly small orthorhombic distortions, as discussed in Section 5 and Figures S8(B-E).

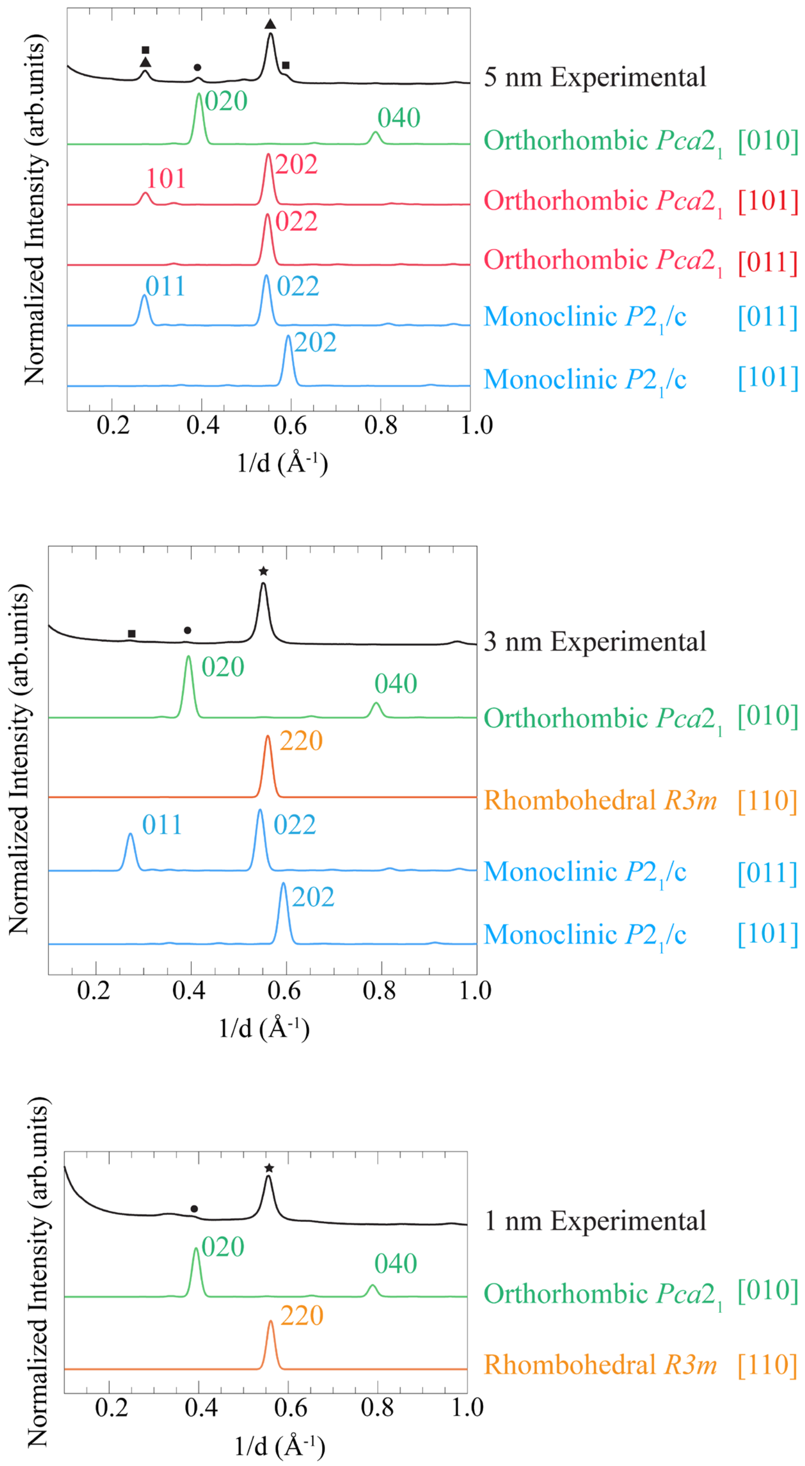

Fig. S10. Reciprocal distance dependence of diffraction intensities for 5-, 3- and 1-nm-thick HZO membranes. Simulated reciprocal distance dependence of diffraction intensities for o-(001), m-(-111), r-(111), and o-(111) are also shown. The sphere, square, star, and triangular marks indicate the diffraction intensity from o-(001), m-(-111), r-(111), and o-(111), respectively.

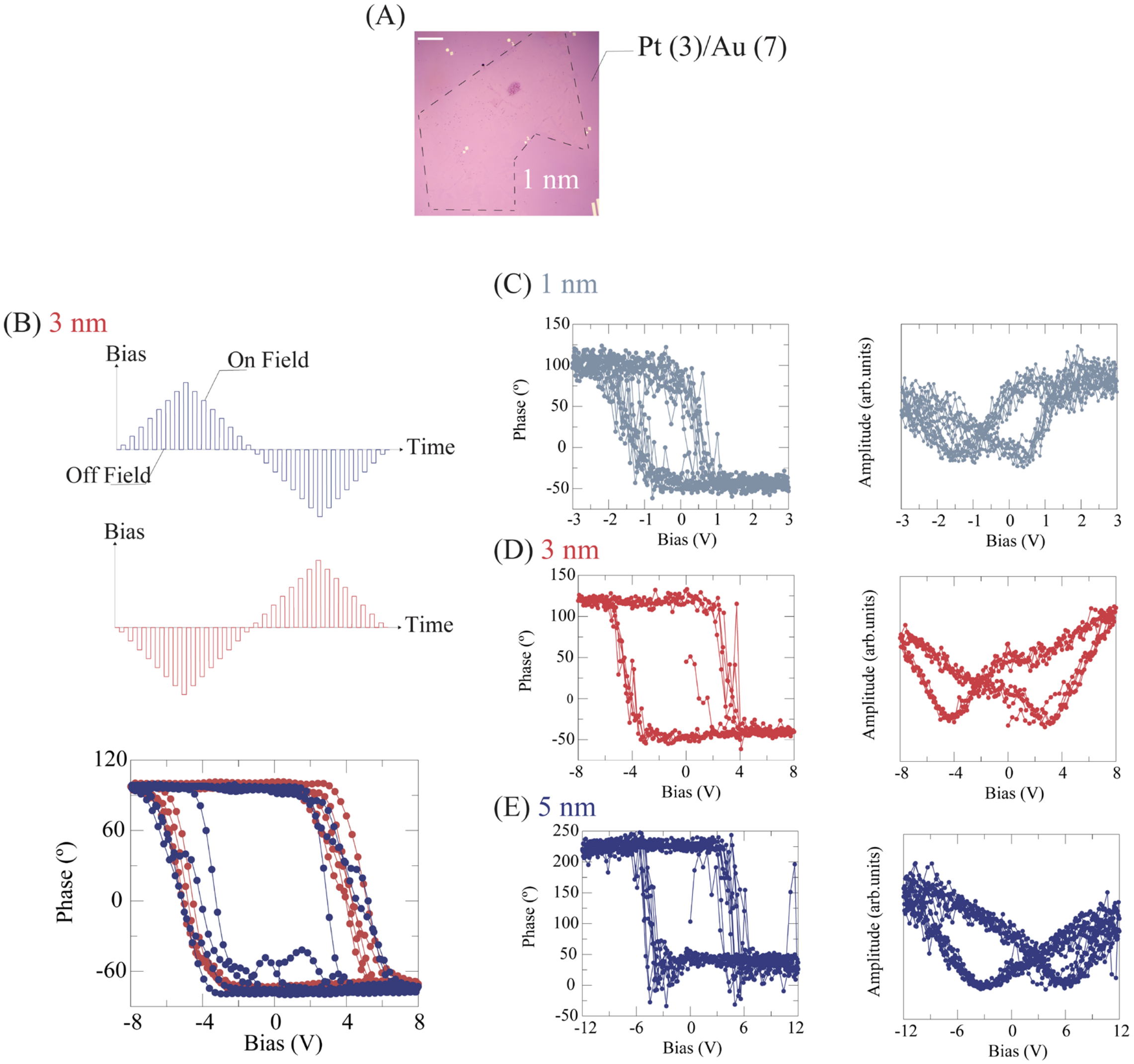

Fig. S11. PFM characterizations of ultrathin HZO membranes. (A) Optical image of the 1-nm-thick HZO membrane transferred onto Pt/Au-sputtered SiO_2/Si substrates. The scale bar in the figure denotes 50 μm. (B) Voltage pulse sequences used for measuring the bias dependence of the out-of-plane off-field piezoelectric response with PFM. The loops show the bias dependence of the off-field PFM phase of the 3-nm-thick HZO membranes, measured in the voltage sweep directions schematically shown in the figure (sweeping from positive to negative or from negative to positive). The loop measurements were repeated four times, and all data were plotted in the figures. (C-E) External bias dependence of out-of-plane off-field PFM phase and amplitude for 1-, 3-, and 5-nm-thick HZO membranes. The measurements were repeated ten times, and all data are plotted in the figure.

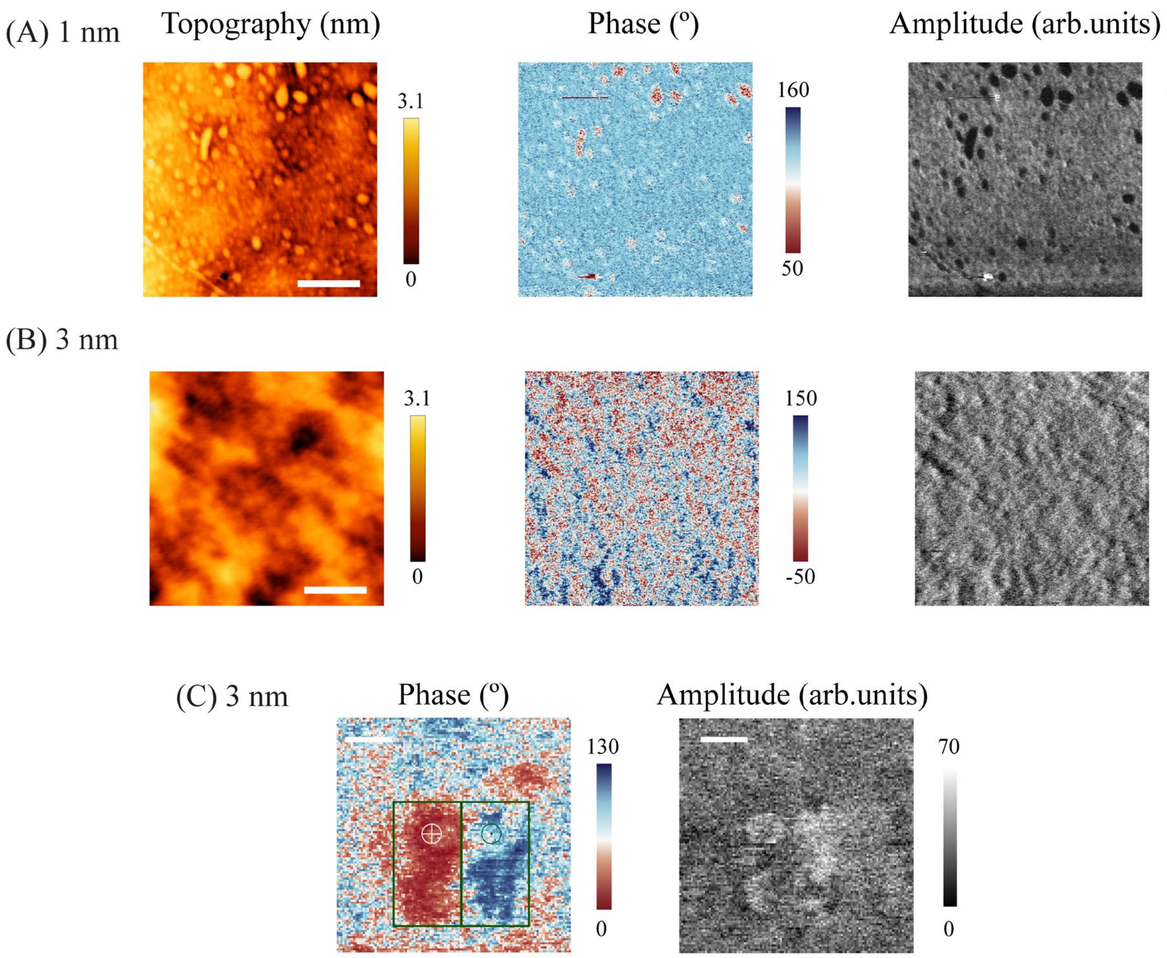

Fig. S12. Ferroelectric domain characterizations of 1- and 3-nm-thick HZO membranes. (A-B) domain mapping of HZO membranes without any poling. The scale bar denotes 500 nm. (C) Switched ferroelectric domains of the 3-nm-thick HZO membranes. The poling bias was ±8 V. The scale bar corresponds to 100 nm.

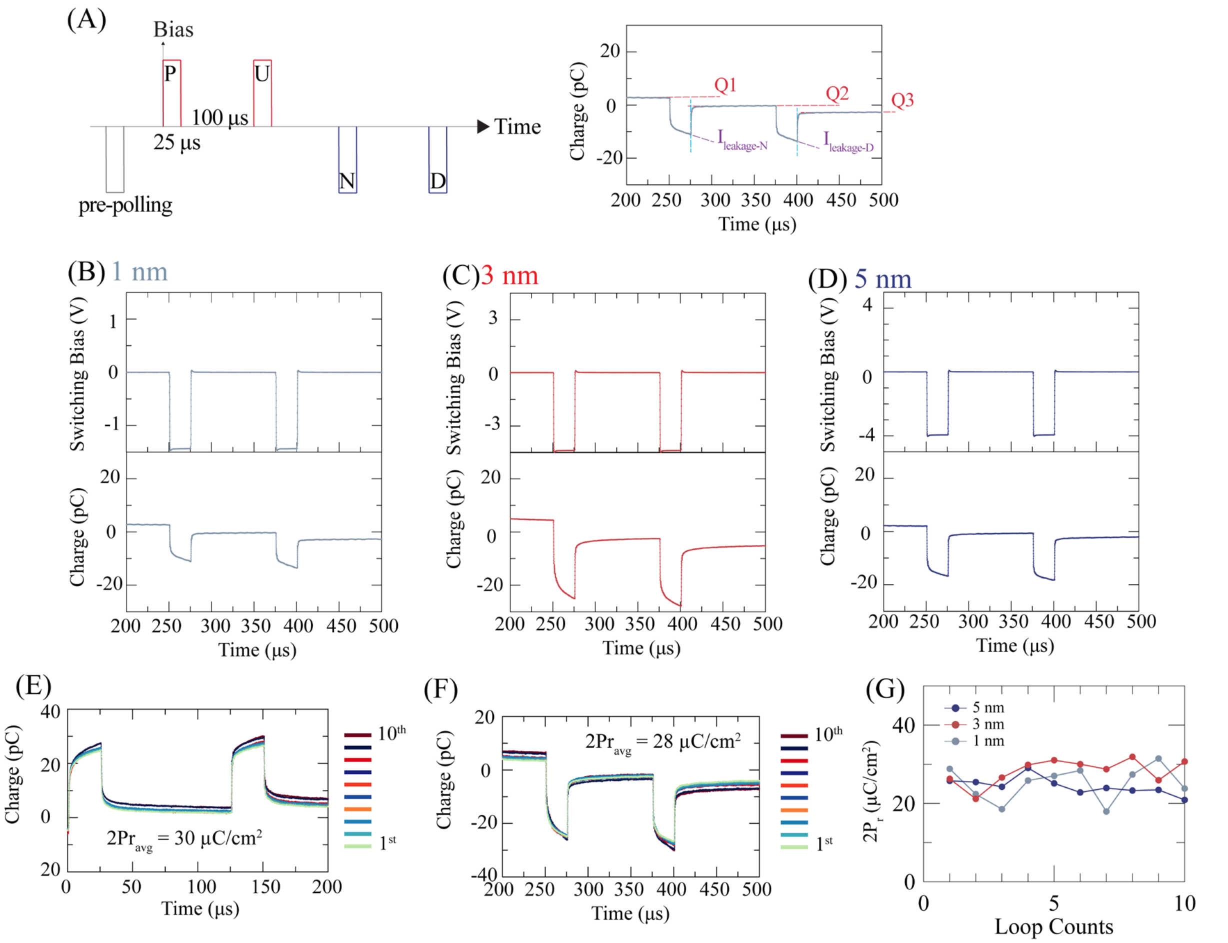

Fig. S13. PUND measurement of ultrathin HZO membranes with different thicknesses. (A) Bias sequences employed in the PUND measurements and typical charge profile obtained. (B-D) Bias sequences and measured charge for 1-, 3-, and 5-nm-thick HZO membranes. (E-F) Changes in the charge associated with polarization switching by (E) positive and (F) negative bias. Data obtained in ten cycles of measurements are plotted. (G) Switchable polarizations for the 1-, 3-, and 5-nm-thick HZO membranes obtained from ten cycles of PUND measurements.

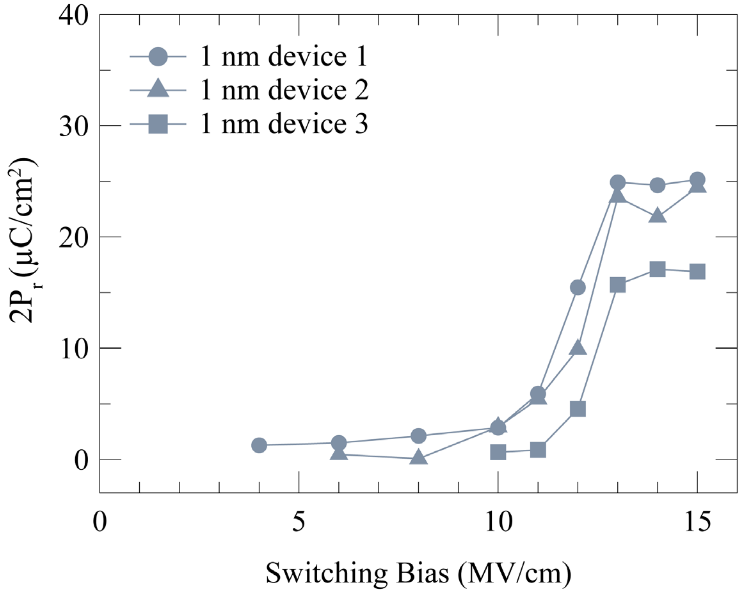

Fig. S14. Switchable polarizations of 1-nm-thick HZO membranes extracted from different cross-bar junctions.

Orthorhombic $Pca2_1$

a=5.04Å b=5.07Å c=5.27Å α=β=γ=90°

	x	y	z	site
Hf0	0.26744	0.24257	0.03379	1a
Hf1	0.26744	0.74257	0.46621	1a
Hf2	0.73256	0.24257	0.53379	1a
Hf3	0.73256	0.74257	0.96621	1a
O4	0.07214	0.37685	0.36791	1a
O5	0.07214	0.87685	0.13209	1a
O6	0.45980	0.49358	0.73329	1a
O7	0.45980	0.99358	0.76671	1a
O8	0.54020	0.99358	0.26671	1a
O9	0.54020	0.49358	0.23329	1a
O10	0.92786	0.37685	0.86791	1a
O11	0.92786	0.87685	0.63209	1a

Monoclinic $P2_1$/c

a=5.11Å b=5.17Å c=5.29Å β=99.211° α=γ=90°

	x	y	z	site
Hf0	0.27514	0.03964	0.20728	4e
O1	0.06600	0.32600	0.33900	4e
O2	0.45600	0.75680	0.48700	4e

Rhombohedral *R3m*

a=b=c=5.05Å α=β=γ=89.98°

	x	y	z	site
Hf1	0.08422	0.58422	0.08422	3b
Hf2	0.58415	0.58415	0.58415	1a
O3	0.30870	0.85972	0.30870	3b
O4	0.81487	0.35390	0.81487	3b
O5	0.85998	0.85998	0.85998	1a
O6	0.35364	0.35364	0.35364	1a

Tables S1. Structural details of the model of the orthorhombic, monoclinic, and rhombohedral phases of HfO_2. The structural parameters of the orthorhombic phase are adopted from the Materials Project database (mp685097). Those for the monoclinic and rhombohedral phases are adopted from the ICSD database (142790) and Ref. 1 in Supplementary Materials, respectively.

1 nm	$I_{leakage}$ (A)		$\Delta Q_{leakage}$ ($\mu C/cm^2$)	$2P_{r\text{-}Corrected}$ ($\mu C/cm^2$) $=2P_{r\text{-}PUND}-\Delta Q_{leakage}$
	-1.14e-07 ± 1.35e-09 (N)	-1.06e-07 ± 1.17e-09 (D)	4.9	24.7
	1.69e-07 ± 1.02e-09 (P)	1.64e-07 ± 1.16e-09 (U)	2.7	27.6
3 nm				
	-1.97e-07 ± 1.81e-09 (N)	-1.73e-07 ± 1.17e-09 (D)	4.7	27.7
	1.48e-07 ± 1.86e-09 (P)	1.33e-07 ± 1.20e-09 (U)	3.0	30.4
5 nm				
	-9.08e-08 ± 8.7e-10 (N)	-8.16e-08 ± 5.85e-10 (D)	5.8	24.7
	9.07e-08 ± 1.55e-09 (P)	8.03e-08 ± 5.18e-10 (U)	6.5	24.9

Tables S2. Leakage current contribution extracted from the charge profiles in the PUND measurements for 1-, 3, and 5-nm-thick HZO membranes. The analysis results for the charge profiles obtained by applying voltage sequences with the largest voltage magnitude (1.5 V, 3V, and 4V for the 1-, 3-, and 5-nm-thick membranes, respectively) are shown.

Supplementary Materials References:

1. Wei, Y. *et al.* A rhombohedral ferroelectric phase in epitaxially strained $Hf_{0.5}Zr_{0.5}O_2$ thin films. *Nat. Mater.* **17**, 1095–1100 (2018).
2. Xu, J. *et al.* A two-dimensional semiconductor transistor with boosted gate control and sensing ability. *Sci. Adv.* **3**, e1602246 (2017).
3. Noori, Y. J. *et al.* Large-Area Electrodeposition of Few-Layer MoS_2 on Graphene for 2D Material Heterostructures. *ACS Appl. Mater. Interfaces* **12**, 49786–49794 (2020).
4. Shen, Y. *et al.* Stabilization of ferroelectric $Hf_{0.5}Zr_{0.5}O_2$ epitaxial films via monolayer reconstruction driven by valence-dependent interfacial redox reaction and intralayer electron transfer. *Phys. Rev. Mater.* **7**, 114405 (2023).
5. Buragohain, P. *et al.* Fluid Imprint and Inertial Switching in Ferroelectric La:HfO_2 Capacitors. *ACS Appl. Mater. Interfaces* **11**, 35115–35121 (2019).
6. Ji, D. *et al.* Freestanding crystalline oxide perovskites down to the monolayer limit. *Nature* **570**, 87–90 (2019).
