# Supplementary material for: Ferroelectric freestanding hafnia membranes with metastable rhombohedral structure down to 1-nm-thick
Source: Nat Commun. 2024 Jun 25;15:4789. doi: 10.1038/s41467-024-49055-w (PMC11199652; doi:10.1038/s41467-024-49055-w)
Supplement: Supplementary file 3 — Lasing Reporting Summary [file 41467_2024_49055_MOESM3_ESM.pdf]

nature research

Corresponding author(s): Daisuke Kan

Lasing Reporting Summary

Nature Research wishes to improve the reproducibility of the work that we publish. This form is intended for publication with all accepted papers reporting claims of lasing and provides structure for consistency and transparency in reporting. Some list items might not apply to an individual manuscript, but all fields must be completed for clarity.

For further information on Nature Research policies, including our data availability policy, see Authors & Referees.

• Experimental design

Please check: are the following details reported in the manuscript?

1. Threshold

Plots of device output power versus pump power over a wide range of values indicating a clear threshold

- [ ] Yes — *State where this information can be found in the text.*
- [x] No — Our research primarily focus on the fabrication and characterization of ferroelectric freestanding hafnia membranes. The laser with fixed wavelength and energy was used only for preparing (La,Sr)MnO3 and (Hf,Zr)O2 films. Parameters such as threshold are not significant.

2. Linewidth narrowing

Plots of spectral power density for the emission at pump powers below, around, and above the lasing threshold, indicating a clear linewidth narrowing at threshold

- [ ] Yes — *State where this information can be found in the text.*
- [x] No — Our research primarily focus on the fabrication and characterization of ferroelectric freestanding hafnia membranes. The laser with fixed wavelength and energy was used only for preparing (La,Sr)MnO3 and (Hf,Zr)O2 films. Parameters such as linewidth narrowing are not significant.

Resolution of the spectrometer used to make spectral measurements

- [ ] Yes — *State where this information can be found in the text.*
- [x] No — Our research primarily focus on the fabrication and characterization of ferroelectric freestanding hafnia membranes. The laser with fixed wavelength and energy was used only for preparing (La,Sr)MnO3 and (Hf,Zr)O2 films and not for special measurements.

3. Coherent emission

Measurements of the coherence and/or polarization of the emission

- [ ] Yes — *State where this information can be found in the text.*
- [x] No — Our research primarily focus on the fabrication and characterization of ferroelectric freestanding hafnia membranes. The laser with fixed wavelength and energy was used only for preparing (La,Sr)MnO3 and (Hf,Zr)O2 films. The coherence and polarization of the emission are not significant.

4. Beam spatial profile

Image and/or measurement of the spatial shape and profile of the emission, showing a well-defined beam above threshold

- [ ] Yes — *State where this information can be found in the text.*
- [x] No — Our research primarily focus on the fabrication and characterization of ferroelectric freestanding hafnia membranes. The laser with fixed wavelength and energy was used only for preparing (La,Sr)MnO3 and (Hf,Zr)O2 films. Parameters such as beam spatial profile are not significant.

5. Operating conditions

Description of the laser and pumping conditions
Continuous-wave, pulsed, temperature of operation

- [x] Yes — We have described the operating condition of the laser used for the film growth in the Methods section.
- [ ] No

Threshold values provided as density values (e.g. W cm^{-2} or J cm^{-2}) taking into account the area of the device

- [ ] Yes — *State where this information can be found in the text.*
- [x] No — We did not provided the threshold as a density value because this parameter is not relevant to our study. But we have added the energy density of the laser fluence during film growth in the Method section.

6. Alternative explanations

Reasoning as to why alternative explanations have been ruled out as responsible for the emission characteristics
e.g. amplified spontaneous, directional scattering; modification of fluorescence spectrum by the cavity

☐ Yes — *State where this information can be found in the text.*

☒ No — Our research primarily focus on the fabrication and characterization of ferroelectric freestanding hafnia membranes. The laser with fixed wavelength and energy was used only for preparing (La,Sr)MnO3 and (Hf,Zr)O2 films. Parameters such as amplified spontaneous and directional scattering are not significant.

7. Theoretical analysis

Theoretical analysis that ensures that the experimental values measured are realistic and reasonable
e.g. laser threshold, linewidth, cavity gain-loss, efficiency

☐ Yes — *State where this information can be found in the text.*

☒ No — Our research primarily focus on the fabrication and characterization of ferroelectric freestanding hafnia membranes. The laser with fixed wavelength and energy was used only for preparing (La,Sr)MnO3 and (Hf,Zr)O2 films. Parameters such as laser threshold, linewidth, cavity gain-loss, and efficiency are not significant.

8. Statistics

Number of devices fabricated and tested

☒ Yes
☐ No

We have fabricated more than 10 pieces of hafnia membranes with different thicknesses by exfoliating different batches of epitaxial films of (Hf, Zr)O2 from substrates, which were deposited separately.

Statistical analysis of the device performance and lifetime (time to failure)

☐ Yes
☒ No

Our research primarily focus on the fabrication and characterization of ferroelectric freestanding hafnia membranes. Although we have confirmed that all data presented in the manuscript are well reproduced, there is no statistical analysis due to no specific devices in this study.
